# Supplementary material for: Jamming-controlled stochasticity in metal-insulator switching
Source: Nat Commun. 2026 Sep 24;17:11082. doi: 10.1038/s41467-026-76844-2 (PMC13696717; doi:10.1038/s41467-026-76844-2)
Supplement: Supplementary file 1 [file 41467_2026_76844_MOESM1_ESM.pdf]

Supplemental Information: Jamming-controlled stochasticity in metal-insulator switching

Nicolò D'Anna,[1,*] Nareg Ghazikhanian,[1] Katherine Matthews,[1] Daseul Ham,[2] Su Yong Lee,[2] Alex Frano,[1] Ivan K. Schuller,[1] and Oleg G. Shpyrko[1]

[1] *University of California San Diego, La Jolla, CA 92093, USA*

[2] *Pohang Light Source-II (PLS-II) Beamline Department, Pohang Accelerator Laboratory, POSTECH, Pohang 37673, Republic of Korea*

[*] ndanna@ucsd.edu

Supplementary Note 1 *Electrical Measurements*

Supplementary Note 1.A *Electrical Measurement setup*

Electrical transport measurements were performed in the four-probe station used for the XPCS measurements with a Keithley source meter 2612B. The results in Supplementary Figure 1 are from the samples studied here by XPCS. Temperature dependence (Supplementary Figure 1a) was measured with a 1 μA current. Thermal switching and associated hysteresis confirm expected device function[1]. Electrical switching (Supplementary Figure 1b) was characterized right before the XPCS experiments, to decide switching currents. The currents I_1 and I_2 in Supplementary Figure 1b indicate multiple switching events where a conductive filament forms. Thus, at 4 mA used during the switching XPCS experiment (Fig. 3 and 4 in main article) the measured device region is fully switched to the On state.

Supplementary Note 1.B *Resistance during XPCS measurements and estimation of heating*

Supplementary Figure 1c shows the voltage measured during the XPCS measurements shown in the main article in Fig. 3 and Fig. 4. The black data in Supplementary Figure 1c corresponds to switching with a 4 mA current at t = 600 s, while the red data is for repetitive On/Off events at 4 mA every 3 s. In both cases, after the initial switching event, the voltage is stable at $\approx 450\ \Omega$ with fluctuations on the order of $\pm 10\ \Omega$. The power in the device is $V \times I \approx 8 \times 10^{-3}$ W. As visible in Supplementary Figure 1a, the sample resistance has a strong dependence on temperature and is thus a good measure of its temperature. To estimate the possible heating of the device, it is useful to compare the slope of the temperature dependence in the resistance vs temperature plot in Supplementary Figure 1a at the experimental temperature and the change in resistance measured during the XPCS experiment in Supplementary Figure 1c. The local slope around 450 Ω in Supplementary Figure 1a is -45 Ω/K, thus the resistance fluctuations of $\pm 10\ \Omega$ correspond to ± 0.5 K, showing that heating is negligible during the experiments.

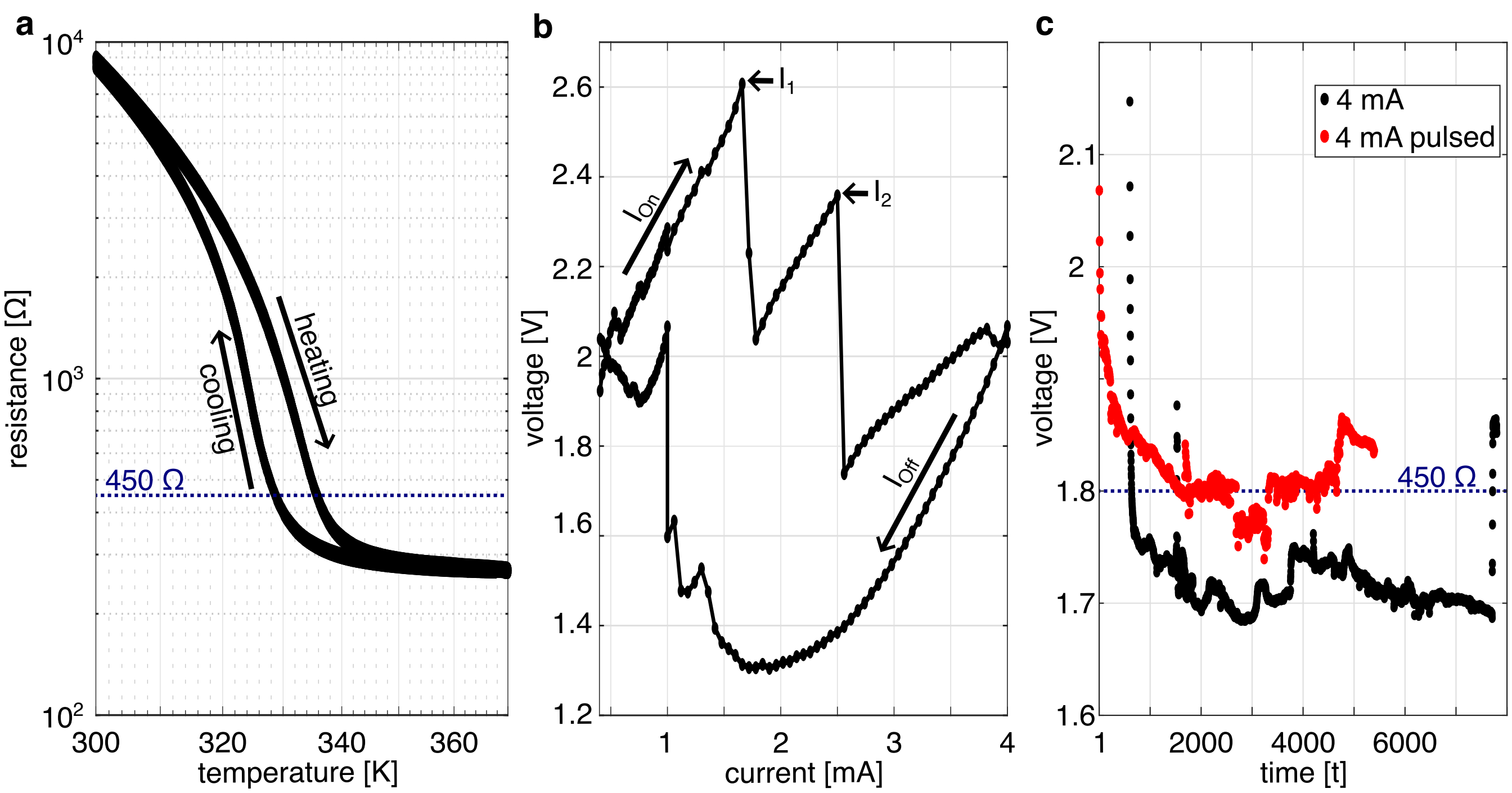

Supplementary Figure 1: **Resistance vs temperature, current, and time.** **a**, Thermal switching measured prior to the XPCS experiment for the device exposed to a 0.1 pC/μm^2 Ga^+ dose. **b**, Electrical switching measured during the XPCS beamtime. I_1 and I_2 indicate the first and second switching currents. **c**, Voltage measured while applying 4 mA current starting at t=600 s (black) and while pulsing the 4 mA current on and off every 3 s (red). The data in **c** were obtained simultaneously with the XPCS data in Fig. 3 of the main article.

Supplementary Note 2 *Bragg diffraction data*

Supplementary Note 2.A *Origin of the Bragg peak and its dynamics*

The diffraction pattern at ≈ 50.5 degrees, shown in Supplementary Figure 2, stems from the insulating 200_{M1} and metallic 011_{R} peaks in VO_2. At 6 keV, the angle separating both peaks is 0.13 degrees[2]. Since the beam size is large (7.4×14.8 μm^2), contributions from both phases overlap on the detector image to create a broad peak. Thus, the percentage of R phase defines the peak center position. Because the diffraction is measured at a high 2θ angle, the sensitivity to changes in the out-of-plane momentum transfer $Q_z = 4\pi \sin\theta/\lambda$ is negligible, with λ the X-ray wavelength. Thus, the diffraction pattern is attributed to the in-plane structure and domains of the VO_2 film, and is described by the kinematic Born approximation; it is the absolute value of the Fourier transform of the in-plane scattering length density. Therefore, the results are not sensitive to thickness features. The center of mass in the 2θ-direction measures the fraction of R phase, while the χ-direction gives the crystal tilt, and the broadening in the χ-direction corresponds to the crystal lattice disorder (or mosaicity). The same Bragg peaks were measured with nanoprobe diffraction[2] to obtain real-space images of the two phases in pristine VO_2 devices equivalent to the ones measured here in terms of fabrication procedure and fabricated by the same group. The nanoprobe imaging showed that the lattice tilt can vary by ±0.11 deg throughout the film, in agreement with Supplementary Figure 2, and that electrical switching nucleates conductive domains of dimension ≈ 100 nm, in agreement with the peak width in the 2θ direction.

For the XPCS analysis, the correlation between subsequent diffraction data is calculated, as explained in the main article, and its decay is fit to obtain timescales of motion. In the case that there are non-dynamic components in the diffraction pattern, as might for example arise from film mosaicity and device structure, their contribution will be included in the absolute value of the correlation $g(t)$, but not in its decay. Thus, the timescales obtained here by XPCS are a measure of the dynamics linked to thermal and electrical switching of domains from the insulating to metallic phase and not from structural properties. At fixed temperatures, despite having reached thermal equilibrium, the dynamics capture thermal motion of insulating and metallic domains, akin to a Brownian motion. Additionally, while the transition observed here is first order, both the insulating and metallic phases are present because defects and inhomogeneity locally impede the transition, resulting in metastable regions (responsible for the hysteresis in Supplementary Figure 1). These metastable regions relax on very long timescales if the material is not annealed and contribute to the observed slow glassy dynamics[3–7]. Finally, the observation of timescales following an Arrhenius-like temperature dependence (inset of Fig. 2d in the main article) is a clear sign that the dynamics are due to physical motion of domains.

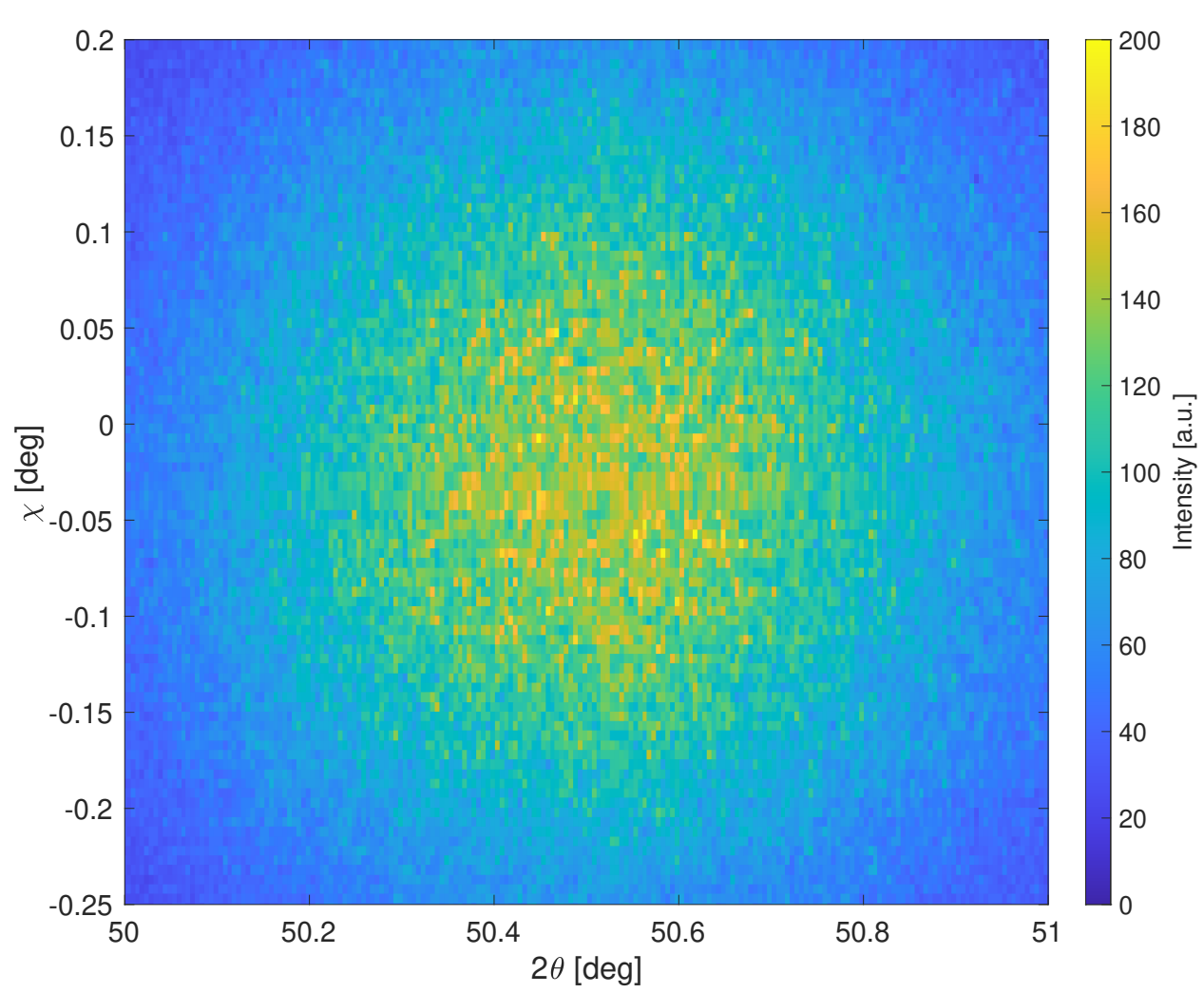

Supplementary Figure 2: **Bragg peak.** Diffraction pattern measured on the irradiated device at 40 °C, with an X-ray energy of 6 keV and detector angle $2\theta \approx 50.5$ degrees.

Supplementary Note 2.B *Time and temperature dependence of the Bragg peak position*

The center of mass position of the Bragg peak during XPCS measurements is shown in Supplementary Figure 3. The green data in Supplementary Figure 3a are obtained on a pristine VO_2 thin layer (corresponding to the data shown in Fig. 2 in the main article), while the blue, red, and black data are obtained when electrically switching the irradiated device and are shown in more detail in Supplementary Figure 3b (and correspond to data in Fig. 3 and 4 in the main article). The change in Bragg peak position induced by heating the thin-layer through the phase transition is ≈ 0.2 degrees, while the change induced by the formation of the conductive filament is ≈ 0.02 degrees. The small change upon electrically switching is due to the X-ray beam being much larger ($7.4 \times 14.8\ \mu\text{m}^2$) than the conductive filament which forms (expected to be on the order of 100 nm^2), such that the majority of the signal stems from the pristine, un-switched region. Nonetheless, the peak center of mass clearly shifts upon electrical switching, Supplementary Figure 3b, showing that the diffraction signal is sensitive to dynamics originating from the conductive filament.

Supplementary Note 2.C *Upper bound on electrical heating*

The observed temperature dependence in Supplementary Figure 3a shows a maximum Bragg peak shift of $\sim$0.2 degrees. In contrast, during the electrical switching in Supplementary Figure 3b, the Bragg peak shifts only by 0.02 degrees, suggesting that global electrical heating of the sample is small. Assuming a linear temperature dependence (0.2 degrees over 60 K), a 0.02 degrees shift would correspond to no more than 6 K electrical heating, considerably larger than the estimation based on the resistance in section 1.B (0.5 K), but small compared to the temperature required for the full thermal transition ($\sim$30 K in Supplementary Figure 1). These considerations are an upper bound, because they neglect the shift induced by the electrically triggered phase transition; the Bragg peak position also reflects the contraction of the lattice as it transitions to the metallic phase, which is independent of the thermal transition. Once the filament is formed, the position of the Bragg peak is constant for the whole measurement time, suggesting that the current is not heating the sample and that the shift in peak position is entirely due to the electrically induced phase transformation. Indeed, a second shift in Bragg peak position would be expected as a result of thermal heating after the fast electrical switching.

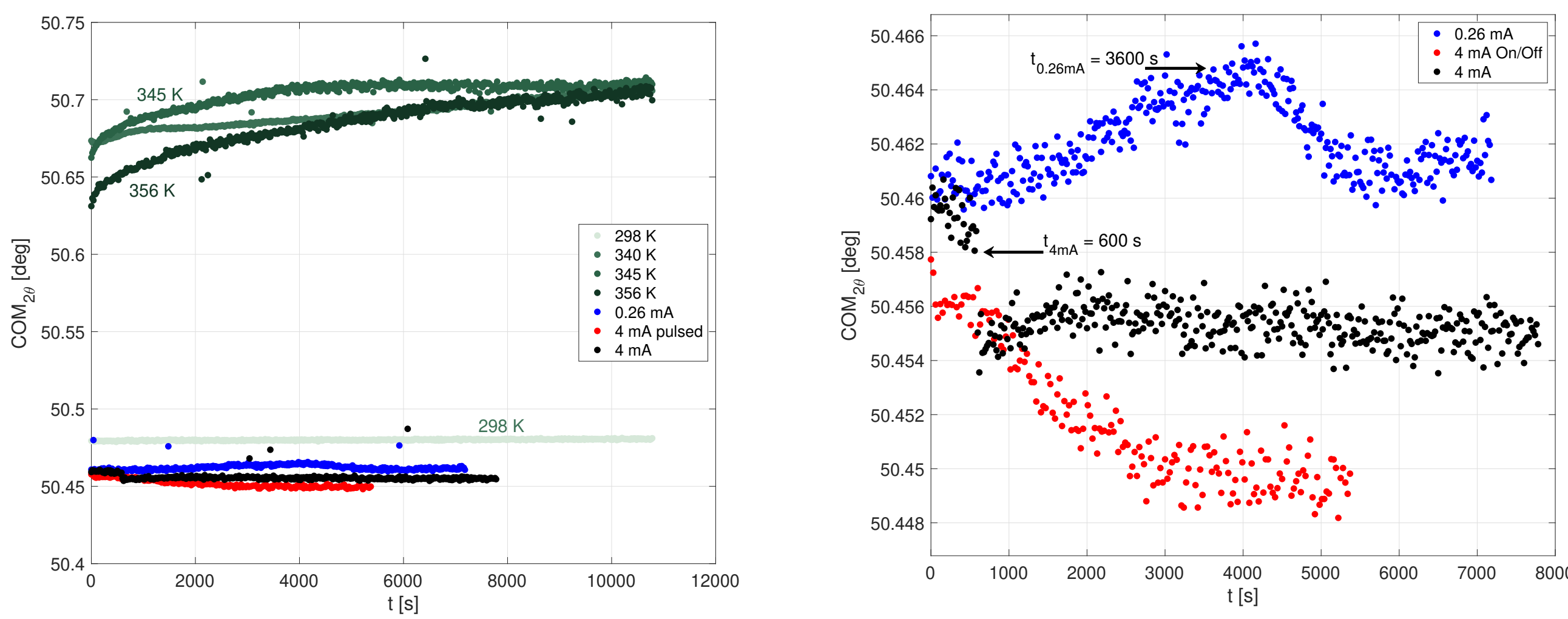

(a) (b)

Supplementary Figure 3: **Bragg peak position during XPCS measurements. a**, Center of mass (COM) of the diffraction Bragg peak on the 2D detector in the 2θ direction for data collected during XPCS measurements. The four green data (light to dark) were taken during the XPCS measurements on pristine VO_2 thin layers shown in Fig. 2 of the main article. The red, blue, and black data were taken during the synchronized electrical and XPCS measurement of the irradiated device in Fig. 3 of the main article. The COM position increases by ~ 0.2 degrees when heating the samples, while it is stable during electrical switching, showing that current-induced heating is negligible. **b**, Detailed plot of the red, blue, and black data in **a**. The COM position shifts when the current is turned on, showing that the measurement is sensitive to the formation of the conductive filament.

Supplementary Note 3 *Results from a pristine device at 60 °C*

Supplementary Note 3.A *Domain dynamics in response to electric fields:*

Correlation decay of sub-threshold (0.24 mA) electrical switching of the pristine device is shown in Supplementary Figure 4a and 4c. It is marked by a slow erasure of the initial nano-domain distribution on timescales ≈ 1000 s, analogous the to the device discussed in the main article.

Repetitive above-threshold (1 mA) electrical switching with a period of 3 s is shown in Supplementary Figure 4b and 4d. The same plastic learning behavior is observed as with the device discussed in the main article. The initial 400 switching events are more stochastic, then gradually become more deterministic until they reach a maximum at ∼ 1000 cycles, corresponding to the maximum correlation observed before switching in Supplementary Figure 4c. These results are equivalent to the ones presented in the main article for an irradiated device, suggesting that in both types of devices switching is governed by the same mechanisms.

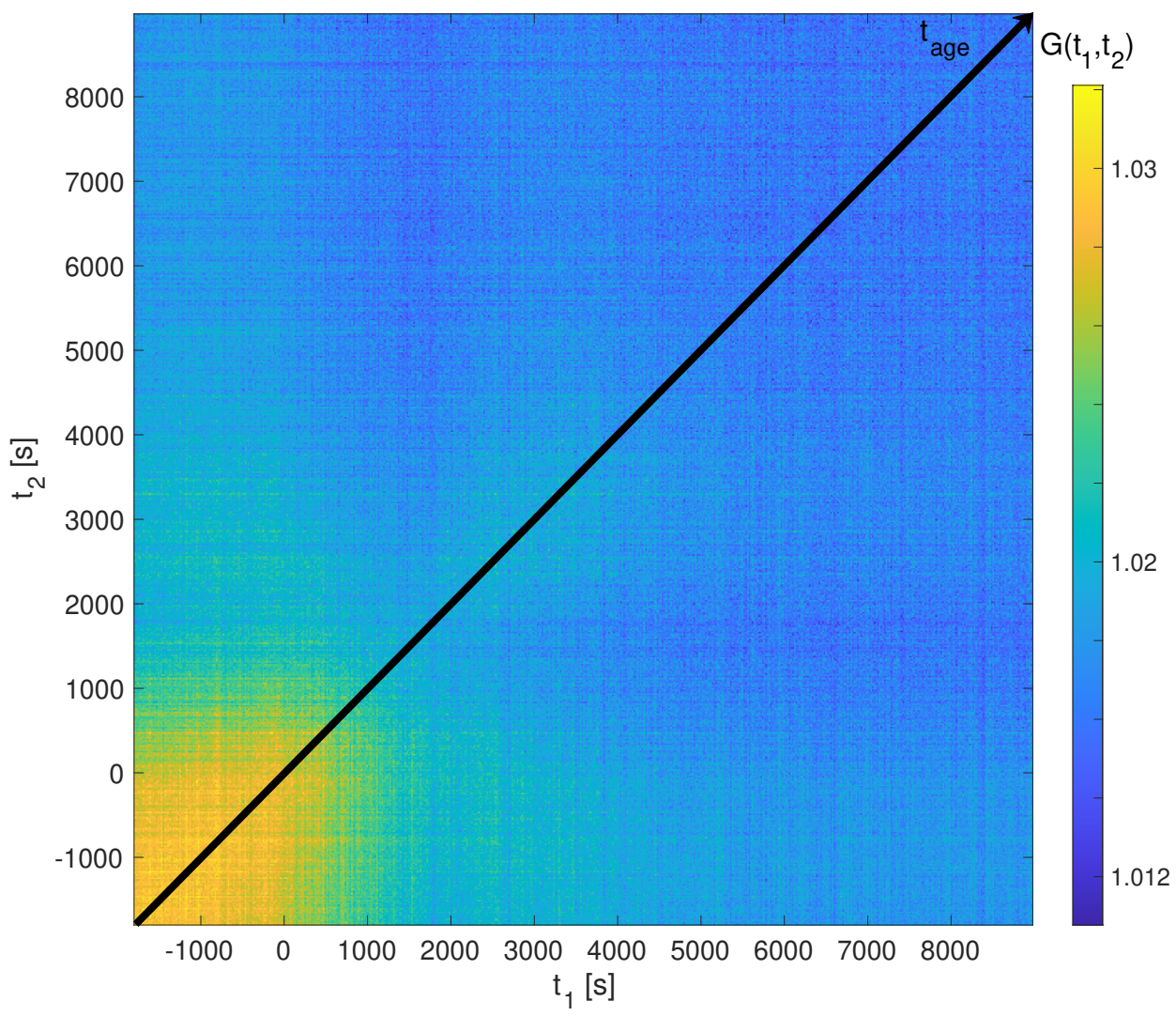

(a)

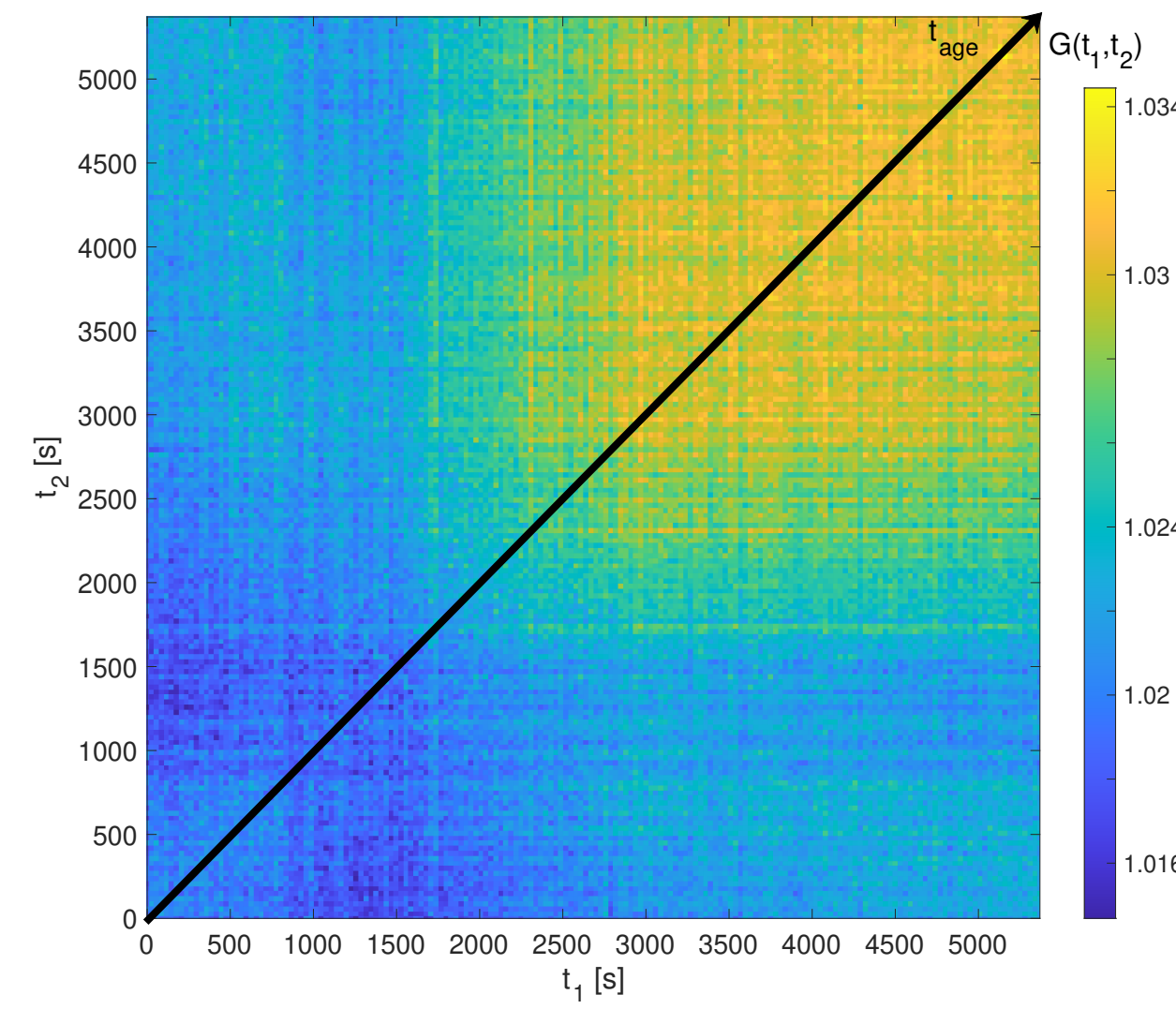

(b)

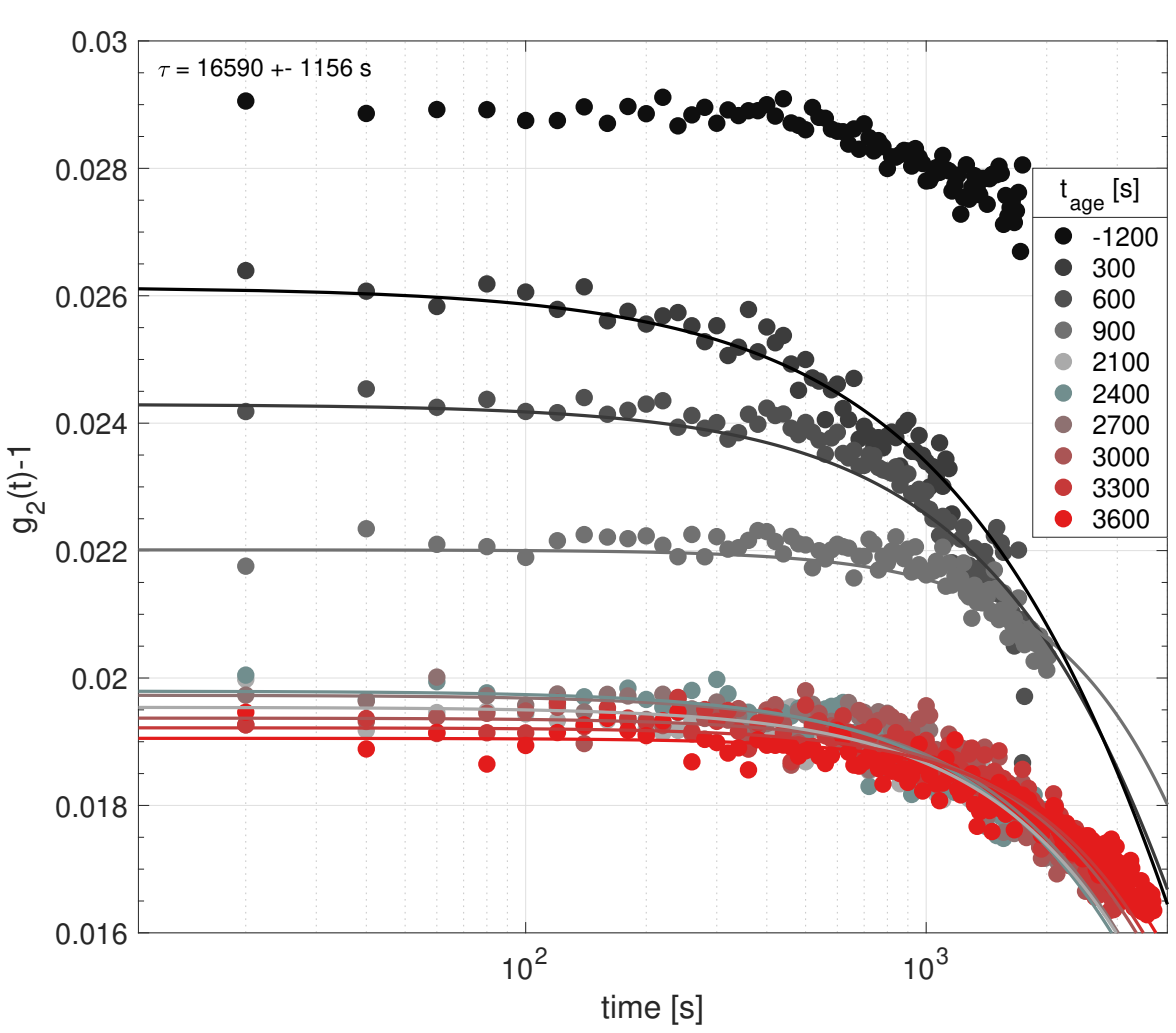

(c)

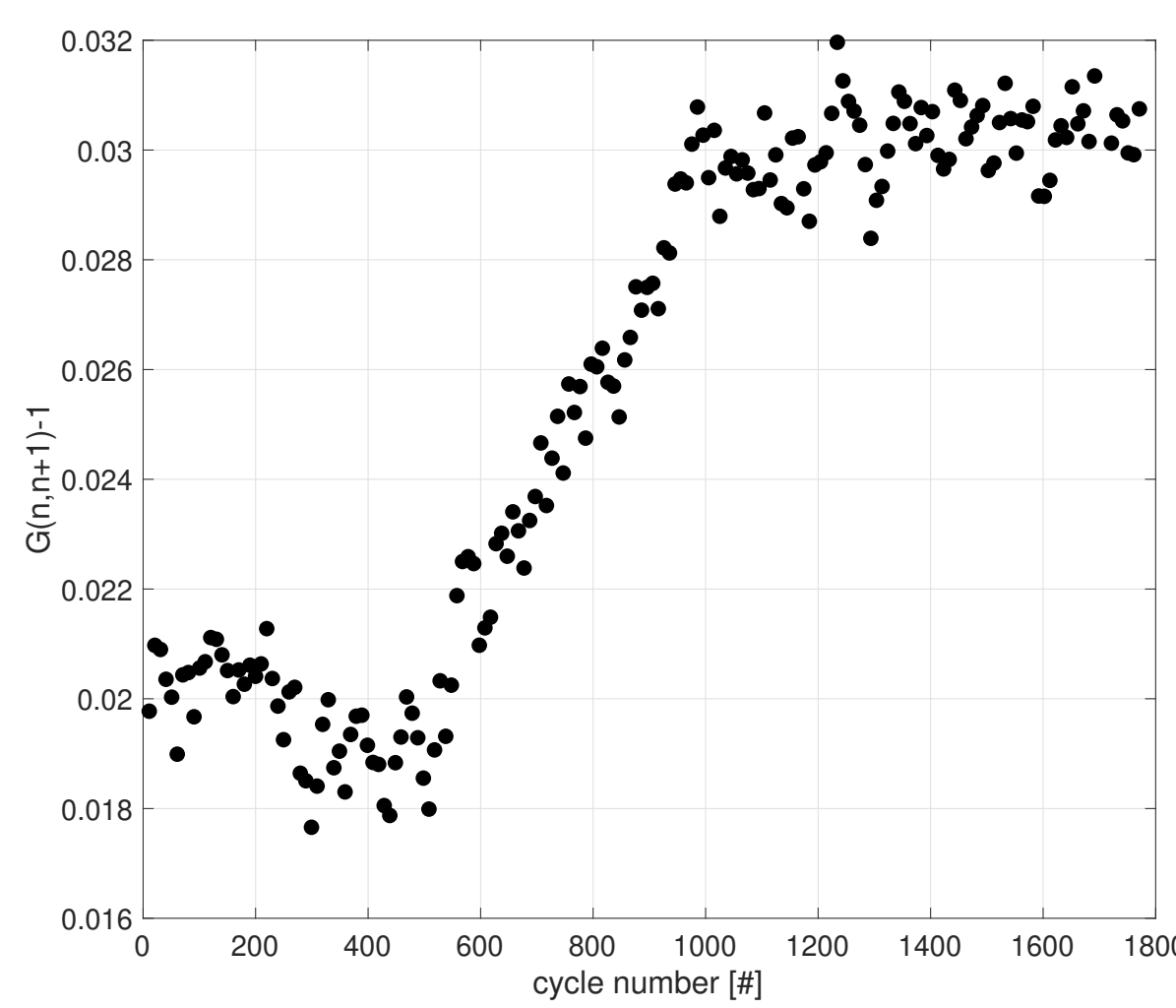

(d)

Supplementary Figure 4: **Electrical switching of a pristine device at 60 °C. a**, Two-times correlation while a 0.24 mA sub-threshold current is turned on ($t = 0$ s). **b**, Two-times correlation while repetitively forming the percolating filament with an above-threshold current of 1 mA. **c**, t_{age} dependent one time correlation $g_2(t)$ obtained from **a**. **d**, Change in correlation $G(n, n+1)$ between cycle n and $n+1$, obtained from the data in **b** normalized by the maximum change.

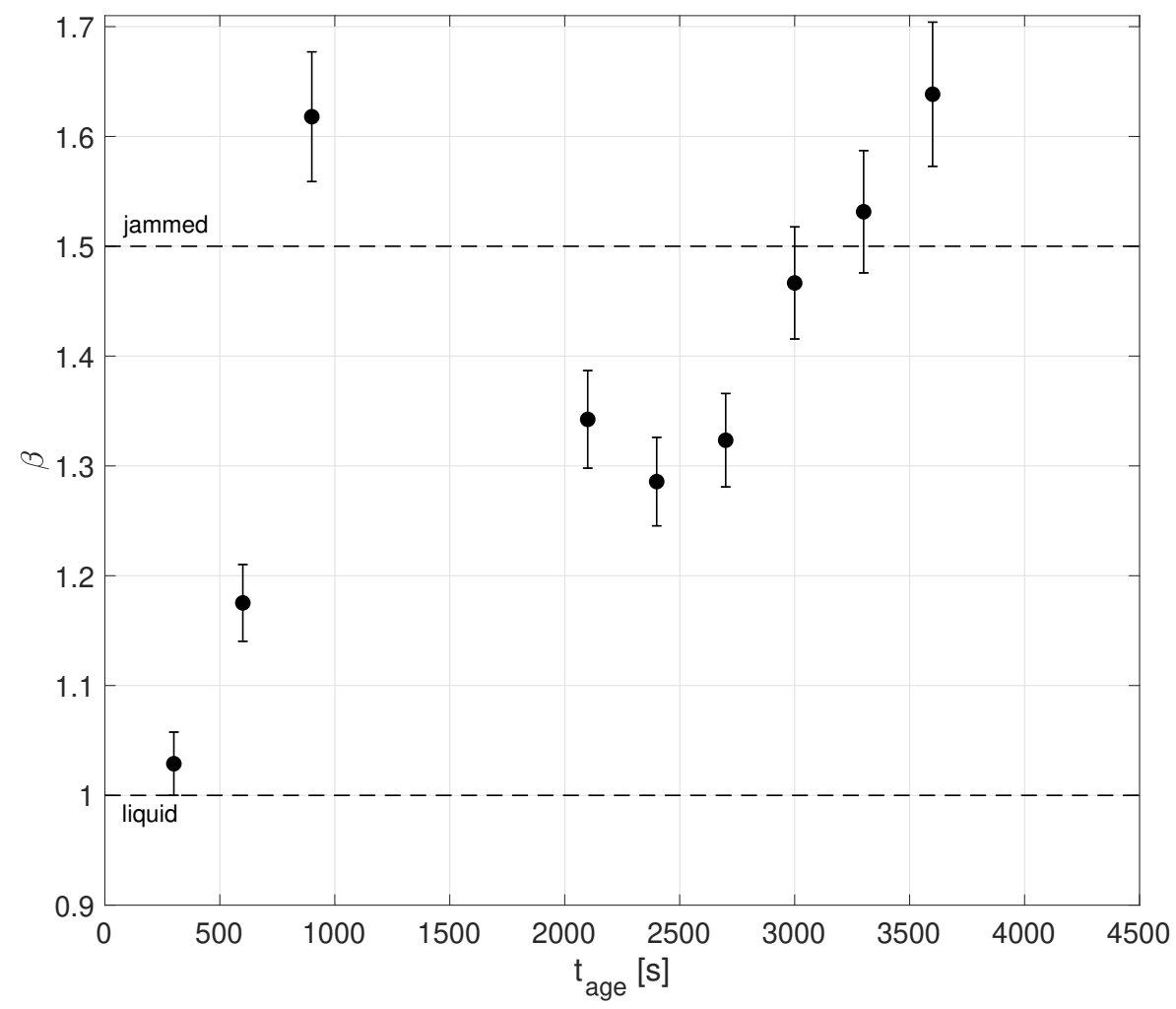

Supplementary Figure 5: **Jamming transition.** Stretching exponent as a function of $t_{\rm age}$, obtained from fits to the data in Supplementary Figure 4c.

Supplementary Note 3.B *Electrically driven jamming transition*

The correlation decay data in Supplementary Figure 4a are fitted with the exponential decay $|F_{t_{\rm age}}(t)| = \exp(-(t/\tau)^{\beta})$. The data for $t_{\rm age} > 4000$ s is not fitted, because the decay was too small to obtain good fits. The stretching exponent β is shown as a function of $t_{\rm age}$ in Supplementary Figure 5. The jamming transition from $\beta = 1$ to $\beta = 1.5$ is also observed in the pristine VO_2 device in the main article.

Supplementary References

[1] Ghazikhanian, N. *et al.* Enhanced stochasticity in irradiated vanadium oxide oscillators. *Advanced Functional Materials* **n/a**, e29777.

[2] Shabalin, A. G. *et al.* Nanoscale imaging and control of volatile and non-volatile resistive switching in VO_2. *Small* **16**, 2005439 (2020).

[3] Samizadeh Nikoo, M. *et al.* Electrical control of glass-like dynamics in vanadium dioxide for data storage and processing. *Nature Electronics* **5**, 596–603 (2022).

[4] Bidoul, N., Roisin, N. & Flandre, D. Tuning the intrinsic stochasticity of resistive switching in vo2 microresistors. *Nano Letters* **24**, 6201–6209 (2024).

[5] Ramírez, J.-G., Sharoni, A., Dubi, Y., Gómez, M. & Schuller, I. K. First-order reversal curve measurements of the metal-insulator transition in vo 2: Signatures of persistent metallic domains. *Physical Review B—Condensed Matter and Materials Physics* **79**, 235110 (2009).

[6] Jo, M. *et al.* Embedded metallic nanoparticles facilitate metastability of switchable metallic domains in mott threshold switches. *Nature Communications* **13**, 4609 (2022).

[7] Del Valle, J. *et al.* Subthreshold firing in mott nanodevices. *Nature* **569**, 388–392 (2019).
