# Supplementary material for: Jamming-controlled stochasticity in metal-insulator switching
Source: Nat Commun. 2026 Sep 24;17:11082. doi: 10.1038/s41467-026-76844-2 (PMC13696717; doi:10.1038/s41467-026-76844-2)
Supplement: Supplementary file 2 [file 41467_2026_76844_MOESM2_ESM.pdf]

nature portfolio

Peer Review File

Jamming-controlled stochasticity in metal-insulator switching

Corresponding Author: Dr Nicolo D'Anna

This file contains all reviewer reports in order by version, followed by all author rebuttals in order by version.

Version 0:

Reviewer comments:

Reviewer #1

(Remarks to the Author)
The manuscript reports the evolution of the nano-domain distribution under several conditions by using X-ray photon correlation spectroscopy. The author observed that defect-engineered VO2 induced by Ga or He FIB irradiation exhibit an ongoing phase transition with a time as long as several thousand second without applied current at its phase transition temperature. Furthermore, they claimed a Jamming transition occurs with a fixed applied current on a time scale about several thousand seconds. Overall, I find the experimental work be interesting and the conclusions are generally well supported by the data and analysis. However, my main concern is the use of defect-engineered VO2 film may affect its novelty and significance. In particular, the RT curves differ markedly between pristine VO2 and defect-engineered VO2 film, especially in terms of the resistance contrast between metallic and insulating states and the width of transition temperature range (Fig. 5 and Fig. S3). These differences suggest that the defected VO2 film difficult to achieve oscillation, which is the key prerequisite for VO2 film for neuromorphic computation. Therefore, although the observations in defected VO2 film look interesting in their own right, it is difficult to say assess to what extent the same physics would also apply to pristine VO2 films.
Below are several comments that may help the authors improve the manuscript:
1. There are several typos in SI, such as “Fig. 1a and ??”, “shown in Fig. ?? and 1d”
2. In the main text, the author said “relaxation to the insulating state may take up to hours for large switching currents”, Why can the relaxation take as long as hours, and under what specific conditions does this occur? In general, relaxation from the metallic to the insulating state is expected to be very fast, typically much shorter than 1 s; otherwise, oscillations in the kHz range would not be possible.
3. In Fig.2b, the author said the XPCS was measured without applying any electric field. If no external stimulus is present, even at transition temperature, why does the film continue to evolve?
4. The author said “The data in the right column, Fig. 2b and 2d, are taken at 340 K, where thermal energy induces domain reconfiguration and increases the amount of metallic domains (the phase transition is expected around 340 K).” Here, Fig. 5 should be cited as direct supporting evidence for the transition temperature around 340 K, rather than relying only on Ref. 18. The transition temperature in VO2 is strongly affected by many factors, including substrate, doping, and defect density. I therefore suggest that Fig. 5 be moved to the Supplementary Information and cited here. More importantly, even if the sample is near the transition temperature, it is still not clear why the film would undergo continuous phase evolution in the absence of an external stimulus. The authors should provide references or further explanation on this point.
5. The author mentioned “a method to control filament location and increase cycling stability is to locally induce defects through Ga or He focused ion beam irradiation of the VO2 thin film”. However, usually, the filament would not be totally random and would appear at several fixed positions during current-induced IMT due to the local defects by strain or from the device fabrication process. On the other hand, if the filament behavior observed here is mainly induced by Ga or He FIB irradiation, then the novelty and broader significance of the work become less clear. The VO2 has attracted considerable interest because of its oscillatory behavior and its potential use as a neuron-like element in neuromorphic devices. If the present study focuses on FIB-induced defects, do these modified films still exhibit oscillations? More importantly, to what extent does this study help understand the behavior in pristine VO2 films?
6. As shown in Fig. 5 and Fig. S3, the R-T curves differ substantially between pristine VO2 and defected VO2 films. In the defected films, the resistance contrast between the metallic and insulating states is less than two orders of magnitude, whereas in pristine VO2 it is close to four orders of magnitude. The transition broadens to about 30-40 K in the defected films, compared with less than 10 K in pristine VO2. Given the substantial difference in transport behavior, it is difficult to conclude that observations made in defected VO2 can be straightforwardly extended to pristine VO2 in the context of

neuromorphic computing. For this reason, the following statement appears overstated: “Regarding neuromorphic computing and emulating neural networks, this gives a possible path forward: a neural network where the connections (synapses) are made with VO2 may be plastically trained by inducing the jamming transition through repetitive switching, and it may be erased by applying a low sub-threshold current.”. Similar to Q5, the author should also have a fair conclusion that to what extent does this study help understand the behavior in pristine VO2 films?
7. Except for the beta value from the fitting, is there any other behavior to support or indicate the Jamming transition?

Reviewer #2

(Remarks to the Author)
The authors report a study of the slow dynamics associated with phase transitions driven by large applied currents in VO2 devices. The transition involves a structural transition and the formation of regions termed domains by the authors that can be probed using X-ray diffraction techniques. The key advance reported by the authors is that the domain dynamics exhibit fluctuations that can be probed by X-ray coherent diffraction techniques that provide statistical insight into the evolution of the domains. Understanding and eventually controlling the switching process in this model system has the potential to inform the design of other systems with eventual device applications and may be of interest to the community. The work also illustrates an expansion of the potential uses of X-ray photon correlation spectroscopy and thus may be of interest to the X-ray science community as well.
I have, however, several concerns that the authors must conclusively address before the validity and scientific utility of the work are sufficiently clear to merit publication:
1) The authors say “the diffraction pattern characterizes the microscopic distribution of the metallic and insulating domains within the X-ray beam.” The manuscript, however, does not establish this connection clearly. The manuscript should include representative diffraction patterns and describe how the distribution of intensity is linked to the domain pattern. Other effects, such as mosaicity, can lead to broadening (and speckle) and need to be ruled out for the manuscript to make the connection compelling to the reader.
2) The manuscript interprets the FWHM of the diffraction peak in terms of the domain size. It is not clear what reciprocal-space direction the authors have used for this analysis or how the domain size should be interpreted in a film with a thickness of 45 nm. Are features associated with the layer thickness observed in the diffraction pattern?
3) One of the subsidiary results reported in the manuscript involves changes in the correlation upon repeated cycling of the device. This result is described intuitively but is not supported by a clear quantitative analysis. Most importantly, the term deltaG is not defined and does not appear to have quantitative meaning. The value of deltaG increases with increasing cycle numbers and is described as an increase in correlation, somewhat counterintuitive for the definitions of deltaG that the reader might guess. The deltaG axis spans 0 to 1 and thus appears to be normalized by an unspecified process that should be discussed in detail.
4) I am somewhat concerned that the work may include significant thermal effects, both in the decorrelation at fixed temperature and the current induced decorrelation. The authors should include an analysis of the current-induced shift in the diffraction wavevector and estimate the resulting current-induced temperature rise – or at least place an upper limit on this quantity. Here it is important to note that the power is high (volts times mA) and is deposited in a thin layer on an insulating substrate. Similarly the authors should illustrate the stability of their apparatus using a thin film sample in the same temperature range. The key issue is that thermal equilibration times can be quite long.
5) Figure 4 relies on the difference between a high-current curve and curves at lower currents. The high current curve includes statistically meaningful points with beta less than one. What is the meaning of those?
6) The authors probe timescales associated with very slow relaxation processes. It is not clear from the work how these timescales are relevant to the switching processes that the authors use to motivate this study. The authors make a vague connection to neuromorphic devices involving training using repeated pulses in the conclusion. The quantitative impact of understanding jamming phenomena in this context – and at this timescale – needs in my opinion to be articulated more clearly.
The manuscript has a few ambiguities and errors that I suggest be corrected:
1) One of the figure references in the supplementary is missing. The text “Fig. 1a and ??.” needs to be corrected.
2) The authors say “diffraction images” when they appear to mean the angular (or reciprocal space) distribution of diffracted intensity. Many readers could find this confusing because diffraction imaging techniques (nanobeam, full-field etc.) are also applied in this system to obtained real-space information.

Version 1:

Reviewer comments:

Reviewer #1

(Remarks to the Author)
The authors have carefully addressed my comments. I agree with the publication in Nature Communications.

Reviewer #2

(Remarks to the Author)
I have reviewed my initial comments and found that the authors have addressed almost all of them. I am puzzled by a few aspects of the response and would like to ask the authors to consider them more carefully. My overall impression remains that the work has the potential to be impactful but that the level of discussion remains qualitative in important places and

should be improved.
1) My original point 3) was asked the authors to make their discussion of Figs. 3g and 3h more precise and to replace or precisely define the quantity plotted there. They have partially done this. But they have also replaced the previous quantity with a new one G(n,n+1) which is similarly not defined in the text. Also F(n,n+1) does not appear to be defined. The precision of the definitions is crucial in establishing the credibility of the manuscript.
2) My point 4) asks the reviewers to consider the effects of heating due to the applied current. Their rebuttal here is highly qualitative and does not provide a clear discussion of the temperature rise induced by the electrical power. The response qualitatively discusses shifts in the angles of reflections but does not connect these to the temperature increase – and in fact confuses the possible heating with shifts due to the phase transition. The supplemental materials seem to indicate that there is a shift in the Bragg angle induced by the electrical current, which could be a possible marker of the phase transition. This point does not necessary precluded publishing the result – but similarly just saying that the qualitative trends are consistent with there being no heating is not going to convince the readers. The response says that the “estimated heating in the sample are now discussed in detail in the SI” but this discussion does not seem to be present.
One additional minor point:
3) The authors have not addressed my question “Are features associated with the layer thickness observed in the diffraction pattern?”.

Version 2:

Reviewer comments:

Reviewer #2

(Remarks to the Author)
The authors have addressed my concerns with sufficient precision that the readers will be able to determine unambiguously what methods were applied. My overall opinion is that the mansucript is now at a level suitable for publication.

Open Access This Peer Review File is licensed under a Creative Commons Attribution 4.0 International License, which permits use, sharing, adaptation, distribution and reproduction in any medium or format, as long as you give appropriate credit to the original author(s) and the source, provide a link to the Creative Commons license, and indicate if changes were made.
In cases where reviewers are anonymous, credit should be given to 'Anonymous Referee' and the source.
The images or other third party material in this Peer Review File are included in the article’s Creative Commons license, unless indicated otherwise in a credit line to the material. If material is not included in the article’s Creative Commons license and your intended use is not permitted by statutory regulation or exceeds the permitted use, you will need to obtain permission directly from the copyright holder.
To view a copy of this license, visit https://creativecommons.org/licenses/by/4.0/

UC San Diego

University of California San Diego

Physics department

9500 Gilman Dr.
La Jolla, CA 92093-0319
USA

Nicolò D'Anna
Postdoc UCSD
Sphyrko group

ndanna@ucsd.edu

Nature Communications Editors

San Diego, 23 May 2026

Response to reviewers

Dear Editor and Reviewers,

Thank you for the clear and in-depth review of our article “Jamming-controlled stochasticity in metal-insulator switching”.

Please find attached a pdf of the revised manuscript, with main modifications and additions in red. The “Supplementary Information” also contains important additional data and analysis.

We have introduced major revisions to the manuscript as suggested by the reviewers. In particular, in-depth discussions about: the origin of the observed speckle pattern and their dynamics, the differences and similarities between irradiated and pristine devices and how our results are relevant to both types of devices and for neuromorphic applications, and, the estimation of current-induced heating and demonstration that it is negligible in the presented results. We also included additional data in the Supplementary Information (3 sections and 3 figures) with in-depth analysis of the sample resistance and Bragg peak center of mass during XPCS measurements, showing thermal stability and the sensitivity of the measurements to the conductive filament formation. Finally, Figure 4i was much improved on the suggestion of Reviewer #2, and Figure 5 with associated Methods section was moved to the Supplemental Information as reviewer #1 suggested.

We would like to thank again the reviewers for their insightful comments, which we have accepted and addressed. Thanks to their help, the revised manuscript is significantly improved. Below is a point-by-point reply to the reviewer comments, with reviewer comments in gray and replies in red.

Yours sincerely,
Nicolò D’Anna on behalf of all authors.

Reviewer #1:
The manuscript reports the evolution of the nano-domain distribution under several conditions by using X-ray photon correlation spectroscopy. The author observed that defect-engineered VO2 induced by Ga or He FIB irradiation exhibit an ongoing phase transition with a time as long as several thousand second without applied current at its phase transition temperature. Furthermore, they claimed a Jamming transition occurs with a fixed applied current on a time scale about several thousand seconds. Overall, I find the experimental work be interesting and the conclusions are generally well supported by the data and analysis. However, my main concern is the use of defect-engineered VO2 film may affect its novelty and significance. In particular, the RT curves differ markedly between pristine VO2 and defect-engineered VO2 film, especially in terms of the resistance contrast between metallic and insulating states and the width of transition temperature range (Fig. 5 and Fig. S3). These differences suggest that the defected VO2 film difficult to achieve oscillation, which is the key prerequisite for VO2 film for neuromorphic computation. Therefore, although the observations in defected VO2 film look interesting in their own right, it is difficult to say assess to what extent the same physics would also apply to pristine VO2 films.
We understand the very valid concern and have added a paragraph in the “Discussion” section regarding the relevance of the results to pristine VO2 and for neuromorphic applications. Equivalent data were obtained in a pristine device, showing that the results are valid for pristine VO2. These are shown in the SI (section III) and mentioned in the “Vanadium dioxide neuromorphic devices” section and in the “Discussion” section. Recent papers on irradiated devices (https://doi.org/10.48550/arXiv.2511.04043, https://doi.org/10.48550/arXiv.2602.14173) show that they also display the relevant oscillatory behavior with increased stochasticity, and that switching energy is reduced by orders of magnitude, making them very relevant for neuromorphic applications. Having obtained equivalent data on both pristine and irradiated devices points to a common switching mechanism.
Below are several comments that may help the authors improve the manuscript:
1. There are several typos in SI, such as “Fig. 1a and ??”, “shown in Fig. ?? and 1d”
We are sorry that many typos were left in the SI. They have now been corrected
2. In the main text, the author said “relaxation to the insulating state may take up to hours for large switching currents”, Why can the relaxation take as long as hours, and under what specific conditions does this occur? In general, relaxation from the metallic to the insulating state is expected to be very fast, typically much shorter than 1 s; otherwise, oscillations in the kHz range would not be possible.
This is a very good point. The statement is supported by https://doi.org/10.1002/smll.202005439 (which is cited in that sentence), where slow relaxation of a full day was observed after the application of a large current (Figure 3). In the same article, after the application of a smaller current, the device quickly relaxes to its initial conductivity state where there is no conductive filament (Figure 2); however, if one looks exactly at the domain configuration, it is visibly still different. If one only looks at the sample resistance (which is an ensemble measurement), the initial state is quickly recovered, but the exact nano-domain configuration can take much longer to be recovered. This behavior is to be expected in a material with such a strong hysteresis loop: the transition is first order, but phase coexistence is observed because of defects and inhomogeneities

pinning domains into metastable, un-switched states. These metastable states eventually relax to the ground state on very slow timescales if not annealed (typical of glassy materials). We also added a citation to this article: https://www.nature.com/articles/s41928-022-00812-z, which shows slow electrical dynamics (recovery and incubation time) in VO2 for up to 10,000 s with features similar to glasses. Finally, we mention these articles: https://doi.org/10.1103/PhysRevB.79.235110, https://doi.org/10.1038/s41467-022-32081-x, https://doi.org/10.1038/s41586-019-1159-6, where persistent metallic domains are observed to remain well below the transition temperature after warming up VO2 samples.

Additionally, XPCS is also sensitive to Brownian-like motion of domains which is not affecting the resistance but is temperature and current dependent. In short, we believe that majority switching is fast and is responsible for the device resistance, but much slower switching of few domains also occurs, and we show that this slow dynamic can influence filament formation stochasticity and be made useful by jamming the state and enabling learning. We now mention these points in the "Vanadium dioxide neuromorphic devices" and "Bragg X-ray photon correlation spectroscopy as a probe of microscale device stability" sections.

3. In Fig.2b, the author said the XPCS was measured without applying any electric field. If no external stimulus is present, even at transition temperature, why does the film continue to evolve?

As mentioned in the previous point, we expect there to be a number of metastable domains which are thermally annealed during these measurements and result in accelerated (but still slow) dynamics. For example, in this article: https://www.nature.com/articles/s41928-022-00812-z, the time it takes for VO2 to fully recover the initial state in terms of switching incubation time after a single switching event is larger than 10,000 s. In addition, some domains might be undergoing thermal fluctuations. The observation of an Arrhenius-like activation energy gives us confidence that the dynamics are meaningful.

4. The author said "The data in the right column, Fig. 2b and 2d, are taken at 340 K, where thermal energy induces domain reconfiguration and increases the amount of metallic domains (the phase transition is expected around 340 K)." Here, Fig. 5 should be cited as direct supporting evidence for the transition temperature around 340 K, rather than relying only on Ref. 18. The transition temperature in VO2 is strongly affected by many factors, including substrate, doping, and defect density. I therefore suggest that Fig. 5 be moved to the Supplementary Information and cited here. More importantly, even if the sample is near the transition temperature, it is still not clear why the film would undergo continuous phase evolution in the absence of an external stimulus. The authors should provide references or further explanation on this point.

These are pertinent points. We agree that the actual transition temperature is determined by the RT measurement, and we added a reference to the figure, which we moved to the SI. We addressed the question about thermal slow dynamics in response to Comment 2 and added a comment in the "Temperature dependence and activation energy in the absence of electric field" section.

5. The author mentioned "a method to control filament location and increase cycling stability is to locally induce defects through Ga or He focused ion beam irradiation of the VO2 thin film". However, usually, the filament would not be totally random and would

appear at several fixed positions during current-induced IMT due to the local defects by strain or from the device fabrication process. On the other hand, if the filament behavior observed here is mainly induced by Ga or He FIB irradiation, then the novelty and broader significance of the work become less clear. The VO2 has attracted considerable interest because of its oscillatory behavior and its potential use as a neuron-like element in neuromorphic devices. If the present study focuses on FIB-induced defects, do these modified films still exhibit oscillations? More importantly, to what extent does this study help understand the behavior in pristine VO2 films?

This is again a good remark. As mentioned in Q1, we added a paragraph in the "Discussion" section regarding the relevance of the results to pristine VO2 and for neuromorphic applications. What is meant by "control filament location" is that while the filament is not completely random in pristine devices, it is difficult to know where it will form prior to the first time switching it, whereas in irradiated devices it will appear in the pre-defined area. Particularly if the VO2 device is large, stochasticity is large as many distinct percolating paths can form [https://doi.org/10.1021/acs.nanolett.4c00184]. In addition, our collaborators recently showed that in irradiated devices the switching takes place in the irradiated area and the relevant oscillatory behavior is seen with a switching energy reduced by orders of magnitude [https://doi.org/10.48550/arXiv.2511.04043, https://doi.org/10.48550/arXiv.2602.14173].

Our view is that in pristine and irradiated devices in the insulating phase there exists a preferential path with lowest resistance due to slightly more conductive domains pinned by defects or strain. When switching, this path will conduct more current and the phase will switch there because of Joule heating. In this picture, the irradiated area makes sure that the lowest resistance path is in the pre-defined area, but the rest of the mechanism is the same. Specifically, the effect is due to higher defect density and not because of Ga or He doping (the ions penetrate to the substrate, leaving only defects in the film). This view is supported by obtaining equivalent data on both pristine and irradiated devices, which points to a common switching mechanism.

We also obtained equivalent data on a pristine device, shown in the SI (section III) and mentioned in the "Vanadium dioxide neuromorphic devices" section and in the "Discussion" section, showing that the results are valid for pristine VO2.

6. As shown in Fig. 5 and Fig. S3, the R-T curves differ substantially between pristine VO2 and defected VO2 films. In the defected films, the resistance contrast between the metallic and insulating states is less than two orders of magnitude, whereas in pristine VO2 it is close to four orders of magnitude. The transition broadens to about 30-40 K in the defected films, compared with less than 10 K in pristine VO2. Given the substantial difference in transport behavior, it is difficult to conclude that observations made in defected VO2 can be straightforwardly extended to pristine VO2 in the context of neuromorphic computing. For this reason, the following statement appears overstated: "Regarding neuromorphic computing and emulating neural networks, this gives a possible path forward: a neural network where the connections (synapses) are made with VO2 may be plastically trained by inducing the jamming transition through repetitive switching, and it may be erased by applying a low sub-threshold current.". Similar to Q5, the author should also have a fair conclusion that to what extent does this study help understand the behavior in pristine VO2 films?

Yes, as mentioned in Q1 and Q5, we have equivalent data for pristine devices in the SI, and added references to recent results showing that irradiated device switching is comparable to pristine devices with the added benefit of much reduced energy consumption and the ability to pre-define filament location through the fabrication process. This is now included in multiple sections.

7. Except for the beta value from the fitting, is there any other behavior to support or indicate the Jamming transition?

The main evidence is the beta exponent, which is a well-accepted feature of XPCS. Another behavior which is consistent with the observed jamming is that we see slow timescales reminiscent of glassy materials, where jamming is common. It is also consistent with the apparent freezing of conductive domains seen in this nanoprobe experiment [https://doi.org/10.1002/smll.202005439], and the observation of persistent domains with glass-like dynamics after switching [https://doi.org/10.1038/s41928-022-00812-z,https://doi.org/10.1103/PhysRevB.79.235110]. These points were added in the "Discussion" section.

Reviewer #2:

The authors report a study of the slow dynamics associated with phase transitions driven by large applied currents in VO_2 devices. The transition involves a structural transition and the formation of regions termed domains by the authors that can be probed using X-ray diffraction techniques. The key advance reported by the authors is that the domain dynamics exhibit fluctuations that can be probed by X-ray coherent diffraction techniques that provide statistical insight into the evolution of the domains. Understanding and eventually controlling the switching process in this model system has the potential to inform the design of other systems with eventual device applications and may be of interest to the community. The work also illustrates an expansion of the potential uses of X-ray photon correlation spectroscopy and thus may be of interest to the X-ray science community as well.

I have, however, several concerns that the authors must conclusively address before the validity and scientific utility of the work are sufficiently clear to merit publication:

1) The authors say "the diffraction pattern characterizes the microscopic distribution of the metallic and insulating domains within the X-ray beam." The manuscript, however, does not establish this connection clearly. The manuscript should include representative diffraction patterns and describe how the distribution of intensity is linked to the domain pattern. Other effects, such as mosaicity, can lead to broadening (and speckle) and need to be ruled out for the manuscript to make the connection compelling to the reader.

This is a very good remark. We have now added a section in the SI (section II) containing an example of the diffraction pattern and a discussion of its origins and the observed dynamics, which is referenced in the main text in the "Bragg X-ray photon correlation spectroscopy as a probe of microscale device stability" section. As the reviewer correctly states, the diffraction pattern on the Bragg peak is the result of all diffraction on the metallic and insulating phases of VO_2. This naturally includes domains of either phase and also structural characteristics, such as the sample geometry, grains, and possible mosaicity. The position of the Bragg peak center of mass gives the amount

of R phase (because both phases have a Bragg peak at almost the same angle, with 0.13 degree separation, less than the peak width), and its width in the chi direction is a measure of the amount of lattice tilting in the device, which can also be due to mosaicity. These values are in agreement with previous studies that we conducted with nanoprobe imaging on similar samples, where we saw that local lattice tilting does not change when switching devices [https://doi.org/10.1002/smll.202005439].

Importantly, in XPCS we only look changes in the diffraction pattern; thus, any static contribution, such as from mosaicity or geometry, does not contribute to the observed de-correlation and timescales. This is why in Figure 1a, when there is no external stimulus or temperature, no dynamics are observed. We only expect phase domains to move with the application of a current or when heating the sample.

In the SI, we also added a part about the time and temperature dependence of the Bragg peak (section II.B), which contains plots of the Bragg peak center of mass angle during the heating and electrical switching. These data are from the same dataset as in Figure 2 and 3 in the main article. What is visible is that when heating the sample, the Bragg peak angle increases, indicating an increase in metallic domains (Figure S3a). When electrically switching (Figure S3b), there is a small but visible shift in the peak angle, showing that we are sensitive to the change of structural phase in the conductive filament, such that the change in correlation observed with XPCS is due to the change in domain configuration induced by the filament formation. We mention this in the main section “Bragg X-ray photon correlation spectroscopy as a probe of microscale device stability” and reference the SI.

2) The manuscript interprets the FWHM of the diffraction peak in terms of the domain size. It is not clear what reciprocal-space direction the authors have used for this analysis or how the domain size should be interpreted in a film with a thickness of 45 nm. Are features associated with the layer thickness observed in the diffraction pattern?

This is a very valid point which should have been discussed in the article. We are only sensitive to the in-plane direction, because the Bragg peak is measured at a high angle where sensitivity to changes in the out-of-plane momentum transfer $Q_z=4\pi\times\sin(\theta)/\lambda$ is very small. We mentioned this in the main article in the first paragraph of “Bragg X-ray photon correlation spectroscopy as a probe of microscale device stability” and in the SI (section II.A).

3) One of the subsidiary results reported in the manuscript involves changes in the correlation upon repeated cycling of the device. This result is described intuitively but is not supported by a clear quantitative analysis. Most importantly, the term deltaG is not defined and does not appear to have quantitative meaning. The value of deltaG increases with increasing cycle numbers and is described as an increase in correlation, somewhat counterintuitive for the definitions of deltaG that the reader might guess. The deltaG axis spans 0 to 1 and thus appears to be normalized by an unspecified process that should be discussed in detail.

Yes, very good point! The normalization was somewhat meaningless (simply max and min). Now we have changed the figure to have two y-axes. The left axis is G(n,n+1) and is plotted with the same y-limits as Figure 3g and 3h for clarity. It shows that the initial correlation is similarly low to when we switch the device once with 4 mA, and that after ~1000 cycles it reaches the same correlation as pre-switching in Figure 3g, which is the by definition the maximum correlation (used to obtain the Siegert factor A in $g_2(t) = 1

+ AIF(t)I^2$). On the right y-axis, we show the intermediate scattering function I F(n,n+1)I^2, which goes from 0 to 1 and corresponds to a percentage of correlation. These new scales are referenced and explained in the main text and in the Methods and make the data on the plot much more meaningful.

4) I am somewhat concerned that the work may include significant thermal effects, both in the decorrelation at fixed temperature and the current induced decorrelation. The authors should include an analysis of the current-induced shift in the diffraction wavevector and estimate the resulting current-induced temperature rise – or at least place an upper limit on this quantity. Here it is important to note that the power is high (volts times mA) and is deposited in a thin layer on an insulating substrate. Similarly the authors should illustrate the stability of their apparatus using a thin film sample in the same temperature range. The key issue is that thermal equilibration times can be quite long.

These are all very valid points. We added two sections in the SI, section I.B and II.B, with 5 figures (S1 and S3) to show that in this experiment, heating is not affecting the XPCS measurements. Section II.B is about tracking the Bragg peak 2θ position throughout the XPCS measurements for temperature and electrical switching. It shows that the change upon electrical switching (where only a small filament is switched) is much less than when fully switching the device thermally, and thus that no significant heating is induced by the current (also seen by the stable peak position after switching). The second section shows the voltage measured in the device during the XPCS scans, where the devices were electrically switched, and compares the fluctuations to the RT to deduce that there are no more than ±0.5 K fluctuations in the device. Additionally, in Figure 2 of the main article, we see that the timescales obtained from the temperature-dependent measurements give us a logical Arrhenius-like activation energy, which tells us that we see the sample heating up and not thermal expansion of the setup. This was expected because we waited for 1 hour before measuring XPCS.

The estimated heating in the sample are now discussed in detail in the SI which is mentioned at the end of the first paragraph in the “Discussion” section and in the second paragraph of the “Bragg X-ray photon correlation spectroscopy as a probe of microscale device stability” section.

5) Figure 4 relies on the difference between a high-current curve and curves at lower currents. The high current curve includes statistically meaningful points with beta less than one. What is the meaning of those?

This is a good point to address. We did not mention these two data points because we felt that their deviation was not significant; however, we now mention them in the main text in the “Electrically driven jamming transition” section. Beta smaller than one can occur when two different timescales with exponent 1 are present, and is consistent with liquid-like behavior.

6) The authors probe timescales associated with very slow relaxation processes. It is not clear from the work how these timescales are relevant to the switching processes that the authors use to motivate this study. The authors make a vague connection to neuromorphic devices involving training using repeated pulses in the conclusion. The quantitative impact of understanding jamming phenomena in this context – and at this timescale – needs in my opinion to be articulated more clearly.

We believe the work is very relevant to device operation at fast repetition rates and have added a paragraph in the “Discussion” section about it. We expect the jamming transition to occur and impact VO2 devices when operating for extended periods of time at kHz, which is necessary for practical neuromorphic computing. Additionaly, as we mentioned in response to Reviewer 1, electrical measurements are an ensemble measurement which is sensitive to the majority of the domains, which switch on very fast timescales. However, after switching, there remains a smaller number of metastable, un-switched domains that have slow dynamics and are important because they define where the conductive filament forms [https://doi.org/10.1103/PhysRevB.79.235110, https://doi.org/10.1038/s41467-022-32081-x, https://doi.org/10.1038/s41586-019-1159-6]. Thus, the observed slow dynamics have an impact on the fast switching. Finally, while we don’t observe the fast switching of the devices, the results will eventually be important and help in finding ways to mitigate or take advantage of the jamming transition, and to determine maximum long-term switching speeds to be used. We also believe that obtaining deterministic switching by purposefully inducing the transition offers an important tuning parameter that is likely to be used for in-device learning.

The manuscript has a few ambiguities and errors that I suggest be corrected:

1) One of the figure references in the supplementary is missing. The text “Fig. 1a and ??.” needs to be corrected.

We are sorry about this oversight and have corrected the broken references and proofread the SI.

2) The authors say “diffraction images” when they appear to mean the angular (or reciprocal space) distribution of diffracted intensity. Many readers could find this confusing because diffraction imaging techniques (nanobeam, full-field etc.) are also applied in this system to obtained real-space information.

This is a good point and we see how it might be confusing. We have now changed it to “diffraction data” instead of “diffraction image“.

UC San Diego

Nature Communications Editors

University of California San Diego

Physics department

9500 Gilman Dr.
La Jolla, CA 92093-0319
USA

Nicolò D'Anna
Postdoc UCSD
Sphyrko group

ndanna@ucsd.edu

San Diego, 16 June 2026

Response to reviewers

Dear Editor and Reviewers,

Thank you for taking the time to evaluate our revisions of our article "Jamming-controlled stochasticity in metal-insulator switching" and to provide further comments.

Please find attached a pdf of the newly revised manuscript and Supplemental Information. The second round of revisions are now in purple, while the first revisions remain in red.

We have introduced additional material for three main points, further discussed in the point-by-point replies on the next page:

1) The definition of the values G(n,n+1) and |F(n,n+1)| are now detailed at the end of the Methods in a new paragraph. For clarity we have also displayed equations in the section "XPCS analysis:" on their own line with equation numbers, which we now reference in the main text.
2) We have complemented the discussions on heating with quantitative estimations of possible heating, showing that heating is negligible for our results. These are based on the resistance measurements, discussed in SI section I.B and yielding a limit of 0.5 K heating, and on the Bragg peak 2θ position, discussed in the new SI section II.C and yielding an upper bound of 6 K.These estimates are upper bounds because they neglect the change in conductivity and Bragg peak position induced by the non-thermal electrical phase transition. These sections are now clearly referenced in the second paragraph of the section "Bragg X-ray photon correlation spectroscopy as a probe of microscale device stability" and at the end of the first paragraph of the discussion in the main article.
3) An explanation of our setup geometry and the reason it is not sensitive to thickness features is now in the first paragraph of the "Bragg X-ray photon correlation spectroscopy as a probe of microscale device stability" and in SI II.A sections. Mainly, because of the large 2θ angle, we are not very sensitive to changes in the out-of-plane Qz-vector.

We would like to thank again the reviewers for their insightful comments, which we have accepted and addressed. Thanks to their help, the revised manuscript is significantly improved. Below is a point-by-point reply to the reviewer comments, with reviewer comments in gray and replies in red.

Yours sincerely,
Nicolò D'Anna on behalf of all authors.

Reviewer #1:
The authors have carefully addressed my comments. I agree with the publication in Nature Communications.
We thank Reviewer #1 for the valuable feedback and for their time.
Reviewer #2:
I have reviewed my initial comments and found that the authors have addressed almost all of them. I am puzzled by a few aspects of the response and would like to ask the authors to consider them more carefully. My overall impression remains that the work has the potential to be impactful but that the level of discussion remains qualitative in important places and should be improved.
1) My original point 3) was asked the authors to make their discussion of Figs. 3g and 3h more precise and to replace or precisely define the quantity plotted there. They have partially done this. But they have also replaced the previous quantity with a new one G(n,n+1) which is similarly not defined in the text. Also F(n,n+1) does not appear to be defined. The precision of the definitions is crucial in establishing the credibility of the manuscript.
It is a good point that these definitions need to be clear for credibility. In the Methods "XPCS analysis" section, we now have the equations defining these quantities. The definition for IF(t1,t2)I is now Eq.3 and g2(t) is Eq.2. Both are standard definitions in XPCS. The value of the intermediate scattering function IF(t)I^2 is the percentage of correlation, which is a quantitative value, related to the autocorrelation function g2 by the Siegert factor A as g2(t) = 1 + A IF(t)I^2. We now added a paragraph in the "XPCS analysis" section defining G(n,n+1) (Eq.4) and IF(n,n+1)I (Eq.5), which are analogous to Eq.1 and Eq.3.
In the main text we now reference Eq.4 and Eq.5 when discussing the data in Figs. 3g and 3h in the "Domain configuration upon repetitive switching: learning by plasticity" section (purple text) and in the figure caption itself. Later in the Discussion, the values of IF(n,n+1)I^2 are used when discussing Figs. 3g and 3h to give quantitative values of the correlation.
2) My point 4) asks the reviewers to consider the effects of heating due to the applied current. Their rebuttal here is highly qualitative and does not provide a clear discussion of the temperature rise induced by the electrical power. The response qualitatively discusses shifts in the angles of reflections but does not connect these to the temperature increase – and in fact confuses the possible heating with shifts due to the phase transition. The supplemental materials seem to indicate that there is a shift in the Bragg angle induced by the electrical current, which could be a possible marker of the phase transition. This point does not necessary precluded publishing the result – but similarly just saying that the qualitative trends are consistent with there being no heating is not going to convince the readers. The response says that the "estimated heating in the sample are now discussed in detail in the SI" but this discussion does not seem to be present.
We apologize that the discussion on heating was not visible. In the SI there are now two sections, I.B and II.C, which discuss heating estimations determined by the electrical measurements and by the Bragg peak center of mass positions, respectively. A clearer reference to these discussions is now found in the last paragraph of "Bragg X-ray

photon correlation spectroscopy as a probe of microscale device stability" and at the end of the first paragraph of the Discussion.

In SI I.B, by comparing the resistance fluctuations during the XPCS electrical switching measurements and the slope of the R vs T data at that resistance (dashed line in Fig. S1a), we conclude that there is no more than 0.5K of temperature fluctuations. This is because if the whole sample was heating due to the current, the resistance would change along the R vs T line. Note that this estimation is an upper bound, because it considers that all the resistance fluctuations are due to heating, while in reality it could be the conductive filament changing dimensions or shape.

In SI II.C, we compare the shift in the Bragg peak 2θ position induced by heating the sample through the transition (~0.2 degrees, Fig. S3a), to the changes observed during the XPCS measurements of electrical switching (~0.02 degrees, Fig. S3a). Again, if we considered that all the change in the peak position during electrical switching was due to temperature effects (neglecting the electrical phase transition), then we would find that the shift in peak position gives an upper bound of 6K heating. This is much less than the temperature needed to induce the full transition (~30 K, Fig. S1a). We also point out that if heating was important, we would expect to see two jumps in the Bragg peak position, one right after electrically switching the device and a second due to the Joule heating induced by the current.

We believe that these bound estimations strengthen the results, as they show that heating effects in the sample can be neglected and we thank the reviewer for having insisted on these points.

One additional minor point:

3) The authors have not addressed my question "Are features associated with the layer thickness observed in the diffraction pattern?".

We are sorry that our reply to the Reviewer's question was not visible, particularly as it is an important clarification to make. The answer is no: because of the large diffraction angle, the sensitivity to changes in the out-of-plane momentum transfer $Q_z = 4\pi\sin\theta/\lambda$ is negligible, meaning that we are not sensitive to thickness features. This is now explained both in the SI II.A section and at the end of the first paragraph of the "Bragg X-ray photon correlation spectroscopy as a probe of microscale device stability" section.
